# Effects of an e-Coach Program on the Knowledge, Attitude, and Practice of Patients Self-administering Their First Insulin Injection: Quasi-Experimental Study

**DOI:** 10.2196/83339

**Published:** 2026-07-10

**Authors:** Mengtian Zhang, Lishuang Zhao, Hui Huang, Zaixiang Tang, Liping Tan, Xiaoyan Zhang, Fengmei Tian

**Affiliations:** 1 Department of Medical Record Statistics, The Second Affiliated Hospital of Soochow University, Suzhou, Jiangsu, China; 2 Department of Endocrinology, The Second Affiliated Hospital of Soochow University, Suzhou, Jiangsu, China; 3 Department of Nursing, The Second Affiliated Hospital of Soochow University, No. 1055, Sanxiang Road, Gusu District, Suzhou, Jiangsu, China, 86 512-67783320; 4 Department of Epidemiology and Biostatistics, School of Public Health, Medical College, Soochow University, Suzhou, Jiangsu, China

**Keywords:** temporal self-regulation theory, e-coach, knowledge, attitude, practice, initial insulin injection

## Abstract

**Background:**

The current level of insulin knowledge, attitudes, and practices of patients self-administering their first insulin injection needs to be improved. There is an emerging need to develop a program for patients self-administering their first insulin injection based on the e-coach model derived from the temporal self-regulation theory.

**Objective:**

This study aimed to examine the effectiveness of a temporal self-regulation theory–based e-coach program on the knowledge, attitude, and practice of patients self-administering their first insulin injection.

**Methods:**

A quasi-experimental research design was used, and the study adhered to the Transparent Reporting of Evaluations with Nonrandomized Designs statement. The study was developed and evaluated in a level IIIA hospital between May 1 and December 1, 2022, in Suzhou, Jiangsu Province, China. The control group received care as usual. The intervention group received a self-regulation program called the e-coach program: the “COACHING” implementation steps. The insulin attitude scores and insulin knowledge-practice scores were compared using repeated-measures ANOVA. In particular, given that baseline blood glucose monitoring frequency differed significantly between the 2 groups, thereby compromising their comparability, a repeated-measures analysis of covariance (ANCOVA) was performed, with monitoring frequency entered as a covariate. This approach was adopted to enhance the scientific rigor of the findings and reduce potential bias.

**Results:**

In total, 86 patients were enrolled; of these, 75 patients were followed up, 40 in the experimental group and 35 in the control group. Repeated-measures ANOVA results revealed statistically significant between-group effects (*F*_1,73_=47.67, *P*<.001), time effects (*F*_2,146_=5.02, *P*=.02), and interaction effects (*F*_2,146_=4.75, *P*=.03) for total insulin knowledge scores in both groups. In both groups, statistically significant between-group effects (*F*_1,73_=16.04, *P*<.001) and interaction effects (*F*_2,146_=12.52, *P*<.001) were observed for total insulin attitude scores, but time effects (*F*_2,146_=2.47, *P*=.11) were not statistically significant. In both groups, statistically significant between-group effects (*F*_1,73_=0.66, *P*=.004) were observed for total insulin practice scores, but time effects (*F*_2,146_=1.05, *P*=.35) and interaction effects (*F*_2,146_=2.82, *P*=.08) were not statistically significant.

**Conclusions:**

The e-coach program based on the TST was effective in improving insulin knowledge and insulin attitude but was not proven effective for insulin practice. A longer follow-up study is needed to uncover its long-term benefits on clinical outcomes.

## Introduction

Diabetes mellitus causes a significant disease burden globally, with an annual type 2 diabetes mellitus cerebrovascular disease mortality rate of 17.15 per 1000 patients. Insulin therapy is effective in improving glycemia, while insulin administration remains underused, and delayed initiation of insulin is still widespread in most countries [1-3]. The high diabetes-specific mortality and morbidity rates worldwide force patients with diabetes to self-administer insulin despite the scarcity of their knowledge, attitude, and practices [4,5].

A survey showed that in terms of insulin injection knowledge, only 12% of patients with knowledge of first-time injection techniques had received training from a diabetologist, and 13% of self-injecting patients reported that they had never received any education related to self-injection, despite the fact that the average time spent injecting was close to 9 years [6], and about 22% of patients did not know how to scientifically adjust their medication [7]. In terms of insulin injection attitude, patients unwilling to use insulin had more negative beliefs than those who were willing to use insulin. The main reason for insulin rejection comes from psychological perceptions: 60.2% of patients have psychological resistance to and fear of insulin injection, and further health education is needed to solve the problem of psychological insulin resistance [8,9]. In terms of insulin injection practice and disease self-management practice, the current situation of patients self-administering their first insulin injection is still not optimistic, the average rate of correct insulin injection skills is only 20% [10].

Health education management can improve the level of patients’ self-management ability; however, although there are various forms of health education, the implementation plan and evaluation strategy of specific studies vary greatly. Furthermore, most of the health education focuses only on insulin injection guidance, and the importance of the education regarding disease awareness and self-management of patients who will be self-injecting insulin for the first time needs to be improved. The e-coach model is a new educational model based on health coaching technology, which is used in chronic disease management to improve lifestyle and mental health. Previous studies have shown that the e-coach health education management model is effective in improving health management, such as promoting physical activity in patients with low back pain through health coaching [11], achieving the goal of 5% weight loss in patients with prediabetes [12], and providing a structured approach to improving weight management in primary care patients with an increased risk of cardiovascular disease in different populations [13].

The study of the influence of individual self-health practices needs to take into account a variety of variables such as time and energy. The temporal self-regulation theory (TST) emphasizes the joint influence of time factors, practical strengths, and self-regulation ability on health practices. According to TST, practical competence results from a complex set of biological, social, and cognitive factors. Behaviors are not always intentional and rational but are influenced by time perspective, behavioral prepotency, and self-regulation capacity. In brief, time perspective refers to the extent to which one considers the future consequences of current behaviors; behavioral prepotency refers to behavioral automaticity (akin to habits), and self-regulation capacity refers to the cognitive ability to monitor and control thoughts, emotions, and behaviors [14].

**Figure 1.**
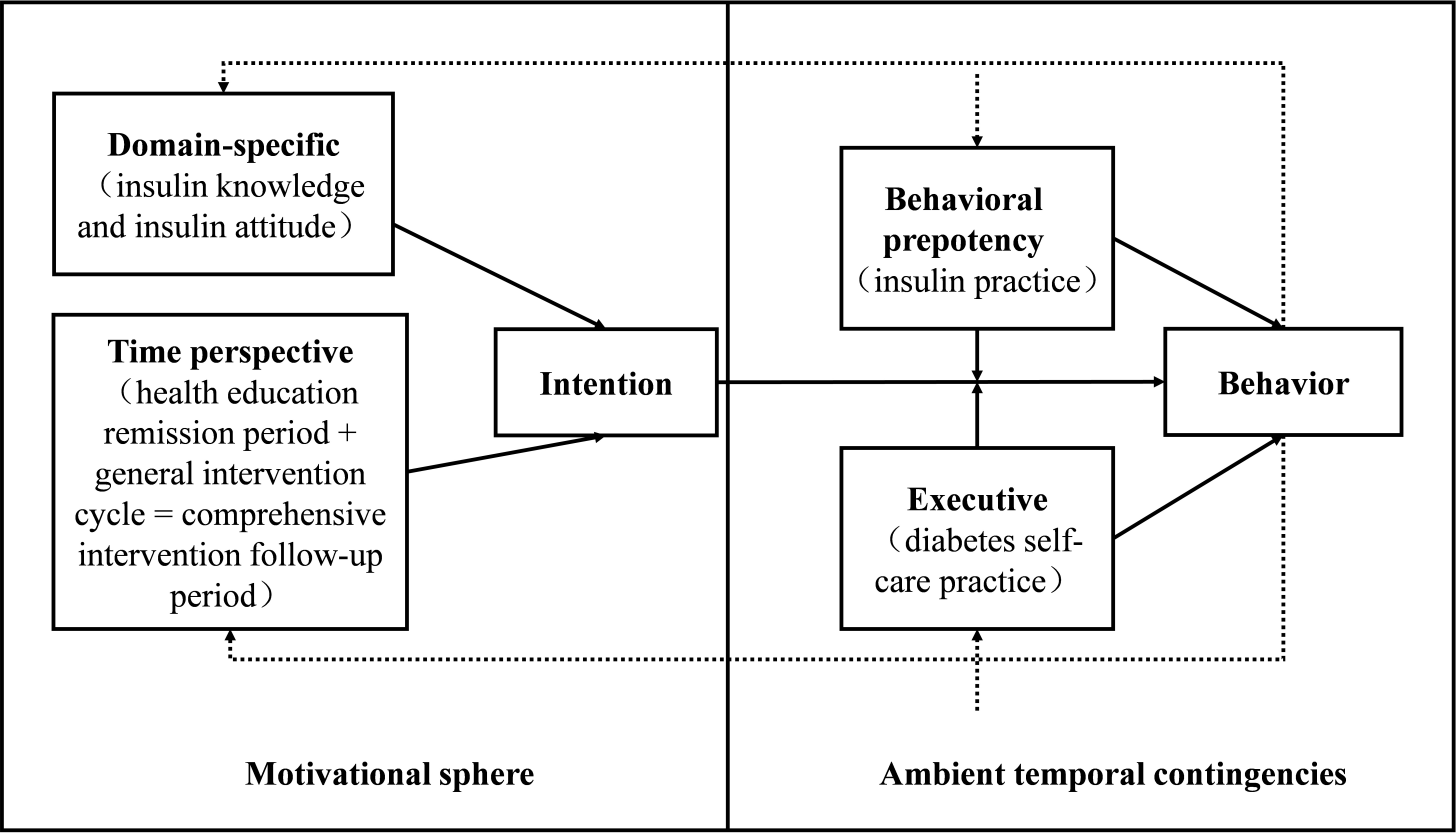
Hypothesized theoretical model of the study.

In this study, TST provided the overall perspective of the e-coach intervention. Since the e-coach model is a model developed from the health coaching program, the general guiding theory of the health coaching program is the theory of planned behavior (TPB) [15], and TPB is a prerequisite for the implementation of interventions based on TST [16], this study used the TST expanded by TPB to guide the overall research process. The specific hypotheses of the study (Figure 1) were that the intervention evaluation indicator variables would be guided by the predictor variables of future behavior in the TST, and the patients’ insulin attitude and insulin knowledge would be used as domain-specific variables in the motivational sphere. The duration of intervention follow-up (at least 3 months), determined by combining the “honeymoon period” for health education among people with diabetes with the standard intervention duration of the e-coach health education program, would be used as a time perspective variable, insulin practice would be used as a behavioral prepotency variable in the ambient temporal contingencies, and self-management practice would be used as an executive variable in the ambient temporal contingencies to comprehensively explore the final self-management ability level of the patients, to understand the corresponding practical orientation of the patients in the future.

The diabetic honeymoon period was initially defined as a transient partial remission phase occurring after diagnosis and initiation of insulin therapy in patients with type 1 diabetes [17]. Given that patients with type 2 diabetes may also experience a remission phase early in the disease course through treatments such as insulin [18], the honeymoon period in this study refers to the time window of significant treatment effect following the first insulin injection in patients enrolled in a diabetes education program. Generally, the duration of the honeymoon period exhibits substantial interindividual heterogeneity and typically lasts from months to years [19].

To the best of our knowledge, there are no intervention studies on the e-coach health education management model using TST as a guiding theory among patients receiving insulin injections for the first time. Therefore, the purpose of this study was to evaluate a TST-guided e-coach health education intervention program using the internet to improve the level of knowledge, attitudes, and practices of patients self-administering insulin injections for the first time and to maintain the effectiveness of health education.

## Methods

### Design and Setting

A quasi-experimental study was conducted to evaluate the effectiveness of a TST-based e-coach program on the knowledge, attitude, practice of patients self-administering insulin injections for the first time. The study adhered to the Transparent Reporting of Evaluations with Nonrandomized Designs statement. Participants provided informed consent during face-to-face interviews with a registered professional nurse for screening and the baseline assessment. Using PASS software (version 15; NCSS, LLC) to calculate the sample size, it was estimated that at least 86 participants were needed to give this study a 90% power at 2-sided 5% level of significance to detect an empirical effect size of 80.7% while allowing for an attrition rate of 20%. Between May 1 and December 1, 2022, 86 hospitalized patients who received their first insulin injection were recruited using convenience sampling at a level IIIA hospital in Suzhou, Jiangsu Province, China.

Repeated outcome measures were collected over 3 time points: before the intervention (T1), month 1 after the intervention (T2), and month 3 after the intervention (T3). Patients were included if they (1) were aged ≥18 years and met the diagnostic criteria of the guideline for the prevention and treatment of type 2 diabetes mellitus in China (2020 edition) [20]; (2) were required to use insulin for the first time under physicians’ orders; (3) had the ability to use smart devices and inject insulin by themselves; and (4) had clear consciousness, no communication barrier, and the willingness to provide informed consent and voluntarily participate in this study. Patients were excluded if they (1) had severe physical disorders or malignant tumors; (2) had severe heart, liver, kidney, or other organ lesions or neuropathy; (3) had mental disorders; (4) dropped out during the study period or did not complete the study for various reasons; or (5) failed to complete the educational course as required.

### Intervention and Control Conditions

#### Control Group: Routine Care

The control group adhered to routine care, while intervention strategies primarily comprised provision of health education upon admission, during hospitalization, before discharge, and through postintervention follow-up phone calls. Upon hospital admission, patients received an orientation based on the protocol, which included familiarizing them with the admission environment, inquiring about their prior history of conventional medication treatment and relevant hospitalizations, and addressing pertinent concerns. During hospitalization, patients were provided with health education on 5 modules: exercise, diet, diabetic foot, hypoglycemia, and drug therapy. It is noteworthy that both the control and intervention groups used the “six-point formula” dietary education handbook [21] for diabetes and “educating patients on diet” instructions displayed in the hospital. Prior to discharge, patients received verbal or demonstrative guidance on insulin injection techniques and were informed that they could consult with diabetes health education nurses at the outpatient clinic for any health education–related inquiries.

#### Intervention Group: e-Coach Program

**Figure 2.**
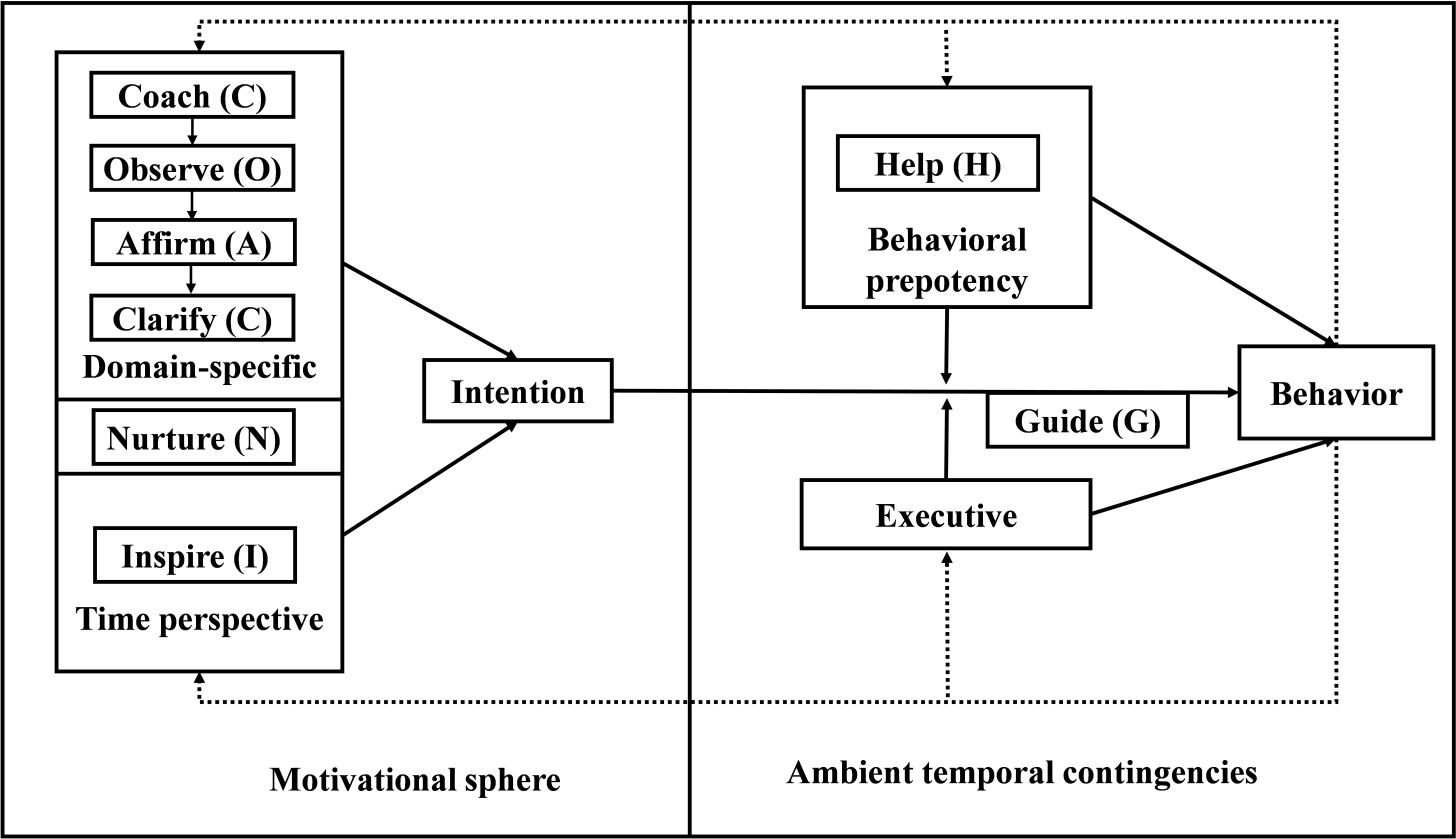
The e-coach program framework guided by the temporal self-regulation theory.

This 3-month program was developed based on the TST, the authors’ previous studies on the local participants’ needs, and a pilot study. Participants assigned to the intervention group received a self-regulation program called the e-coach program; the specific “COACHING” implementation steps are shown in Table 1 and Figure 2.

**Table 1.** Description of the components of the TST^a^-based e-coach program.

Session and topic	Form	Description
Coach (C)	Offline	Patients are guided to develop a domain-specific understanding of TST and construct a cognitive understanding of insulin and diabetes management.
Observe (O)	Online	Patients are assisted to retain a domain-specific understanding of TST while gradually gaining knowledge about administering insulin and managing diabetes.
Affirm (A)	Online	This stage improves the domain-specific understanding of TST and empowers patients to enhance their knowledge of insulin management and diabetes care.
Clarify (C)	Online	This stage upholds the domain-specific TST concept and empowers patients to maintain their understanding of insulin and diabetes management.
Help (H)	Online and offline	This stage establishes the behavioral prepotency of TST to enable patients to maintain insulin and diabetes self-management practice.
Inspire (I)	Online and offline	This stage guarantees the temporal orientation in TST and facilitates the seamless fulfillment of the intervention cycle.
Nurture (N)	Online	This stage leverages the comprehensive effect of the domain-specific and time perspectives in TST and the educational content involved gradually improves the patient’s behavioral prepotency and self-regulation ability.
Guide (G)	Online	This stage provides the comprehensive guidance of TST behavioral prepotency and executive variables to guide patients to improve their self-management ability.

a TST: temporal self-regulation theory.

### Outcome Measures

#### General Characteristics

The study participants self-reported demographic characteristics (name, age, sex, contact information, marital status, place of residence, educational level, occupation, monthly income, height, weight, and blood pressure) and disease-related characteristics (course of diabetes, type of diabetes, treatment of diabetes, frequency of blood glucose monitoring, type of the first insulin therapy, times of injection, injection dose, and complications) using the structured assessment questionnaire developed by the research team and personal interviews conducted individually to protect each participant’s privacy.

#### Insulin Use Knowledge-Practice Scale

The insulin use knowledge-practice scale [22] is mainly used to understand patients’ knowledge and practice related to insulin use, including the 2 dimensions of knowledge and practice. The number of items were 9 and 7, respectively, a total of 16 items. Each item in the knowledge dimension is a single-choice question, with 1 point for the correct answer, 0 points for the wrong answer or no answer, and a maximum possible total score of 9. The higher the score, the better the knowledge mastery. The 7 items in the practice dimension were scored on a 5-point Likert scale, with a maximum possible total score of 35. The higher the total score, the more standardized and correct the practice. The Cronbach α of the scale was 0.76. The total content validity index of the questionnaire was 0.89 for insulin use knowledge and 1 for insulin use practice. The reliability and validity of the scale were good.

#### Insulin Attitude Scale

The insulin attitude scales are derived from the first part of the “insulin use interview tool” developed by the Chinese Diabetes Society [23], which is used to evaluate the attitude and cognition of patients with insulin injections. The tool consists of 2 parts: the main scale and the secondary scale. The main scale is adapted from the Chinese version of the international Diabetes Attitude Wishes and Needs (DAWN) study and contains 20 items. The secondary scale was developed according to the survey report on the current situation of self-management and influencing factors of Chinese patients with type 2 diabetes, including 7 items. The scale contains 7 dimensions and a total of 27 items, scored on a 5-point Likert scale. The lower the total score, the greater the patients’ worry about insulin use and cognitive impairment. The Cronbach α of the scale was 0.85 and CVI was 0.75 [24].

### Ethical Considerations

The trial was registered in the Chinese Clinical Trial Registry (ChiCTR2200058895) and conformed to the ethical guidelines of the 1975 Declaration of Helsinki. All study participants provided informed consent, and ethics approval was obtained from the Ethics Committee of the Second Affiliated Hospital of Soochow University (protocol code JD-LK-2022-025-01). It is worth noting that the registered sample size differs from the actual sample size due to variation in the sample size calculation tool version and the reference used. The sample size was reassessed using more recent tools, and the post hoc power analysis confirmed a statistical power exceeding 80%. Specifically, a power calculation was performed using PASS 15 software, based on a 2-sample *t* test, with the α set at .05 (2-tailed) and an effect size of Cohen *d*=0.426. Given 40 participants in the intervention group and 35 in the control group, the achieved statistical power was 80.7%, meeting the conventional threshold of >80% for statistical power. Furthermore, after the effectiveness of the experimental group’s protocol has been validated, the e-coach management program will be implemented on the control group based on their consent, ensuring the accuracy of the principle of fair compensation.

### Statistical Analysis

Using Microsoft Excel 2019 to establish a database and double-entry data, we numbered the study data and established different databases according to different scales to ensure the accuracy of the study data. SPSS software (version 26; IBM Corp) was used to statistically analyze the data, and the difference was considered statistically significant when *P*<.05. GraphPad Prism software (version 8.0; GraphPad Software) was applied to plot and analyze the repeated measures data.

Descriptive statistical analyses were performed on the general characteristics: among them, the measurement data conformed to normal distribution and were expressed using mean (SD); the count data were expressed using frequency and percentage. The baseline data of the 2 groups of patients were analyzed to verify whether they conformed to normal distribution and compared using *t* test and chi-square test.

Insulin attitude scores and insulin use knowledge-practice scores at all 3 time points were found to be normally distributed. Changes in the corresponding score outcome metrics and treatment effects across time points were then compared using repeated-measures ANOVA. In particular, given that the baseline blood glucose monitoring frequency differed significantly between the 2 groups, thereby compromising their comparability, a repeated-measures analysis of covariance (ANCOVA) was performed, with monitoring frequency entered as a covariate. This approach was adopted to enhance the scientific rigor of the findings and reduce potential bias.

## Results

### Recruitment

A total of 86 participants were enrolled in this study. Owing to the study procedures and various contributing factors, 3 of 43 (6.98%) participants in the intervention group and 8 of 43 (18.60%) participants in the control group were lost to follow-up. Overall, 11 of 86 participants were lost to follow-up, corresponding to an overall attrition rate of 12.79%. During the follow-up period, 5 participants were lost because of nonadherence to the prescribed insulin dosage (2 in the intervention group and 3 in the control group). In addition, 1 participant in the control group self-discontinued insulin therapy. An additional 5 participants were lost to follow-up because they could not be reached by telephone or WeChat (1 in the intervention group and 4 in the control group). Ultimately, 75 participants successfully completed the follow-up: 40 in the intervention group and 35 in the control group. To verify that loss to follow-up did not introduce bias into the final analysis, we first compared the baseline demographic and clinical characteristics between participants who completed the study and those lost to follow-up within each group separately and observed no statistically significant or clinically meaningful differences (Table S1 and Table S2 in Multimedia Appendix 1). Furthermore, comparisons of the numbers and rates of loss to follow-up between the groups showed no statistically significant difference (*P=0.20*), indicating that the participants lost to follow-up would not affect the study results and that comparative analyses were appropriate (Table 2). We also compared the characteristics of the 75 participants who completed the study across the 2 groups and found that the characteristics were well balanced, confirming that the final analytical sample remained representative of the original study population.

**Table 2.** Comparison of loss to follow-up between the experimental and control groups^a^.

Group	Lost to follow-up, n	Completed, n	Total, n	Attrition rate, %
Experimental	3	40	43	6.98
Control	8	35	43	18.60
Total	11	75	86	12.79

a Fisher exact test was used to compare the difference in attrition between groups; *P=0.20*.

### Sample Characteristics

Table 3 presents the sociodemographic and health-related characteristics of all 75 participants. With the exception of the frequency of blood glucose monitoring, the 2 groups had comparable clinical characteristics and outcome measures. The comparison of the number of participants lost to follow-up and the attrition rate between groups showed no statistically significant difference (*P*=.05 to .97). This indicates that the differential impact of attrition on the study outcomes was not statistically significant, thereby supporting the validity of comparative analyses (Table 3).

**Table 3.** Baseline sociodemographic characteristics, clinical profile, and outcome variables of the participants (N=75).

Characteristics	Total (N=75)	Intervention group (n=40)	Control group (n=35)	Chi-square (*df*)/*t* test (*df*)	*P* value
Sex, n (%)				0.2 (1)	.83
Male	57 (76)	30 (75)	27 (77.14)		
Female	18 (24)	10 (25)	8 (22.86)		
Age (years), mean (SD)	43.32 (12.73)	44.85 (11.96)	41.57 (13.52)	1.11 (73)	.27
Height (cm), mean (SD)	170.21 (8.05)	169.38 (8.18)	171.17 (7.90)	−0.96 (73)	.34
Weight (kg), mean (SD)	76.04 (17.35)	72.05 (16.50)	80.61 (17.40)	0.018 (73)	.90
BMI, mean (SD)	26.06 (4.68)	24.96 (4.60)	27.32 (4.50)	0.095 (73)	.76
BP^a^ (mm Hg), mean (SD)					
Systolic	129.36 (19.62)	127.43 (16.92)	131.57 (22.36)	−0.91 (73)	.36
Diastolic	85.37 (13.96)	82.48 (12.46)	88.69 (15)	−1.96 (73)	.054
Marital status				−1.12 (1)	.26
Single	17 (22.67)	7 (17.50)	10 (28.57)		
Married	58 (77.33)	33 (82.50)	25 (71.43)		
Monthly income (¥)^b^, n (%)				5.7 (2)	.06
<3000	8 (10.67)	6 (15)	2 (5.71)		
3000‐5000	10 (13.33)	8 (20)	2 (5.71)		
>5000	57 (76)	26 (65)	31 (88.57)		
Education level, n (%)				1.6 (2)	.46
Junior high school and below	26 (34.67)	16 (40)	10 (28.57)		
Senior high school or college degree	29 (38.67)	13 (32.50)	16 (45.71)		
Bachelor degree or above	20 (26.66)	11 (27.50)	9 (25.72)		
Occupation, n (%)				9.5 (4)	.05
Worker or farmer	11 (14.67)	9 (22.50)	2 (5.70)		
Staff	31 (41.33)	15 (37.50)	16 (45.71)		
Retiree	9 (12)	6 (15）	3 (8.57)		
Student	1 (1.33)	1 (2.50)	0 (0)		
Other	23 (30.67)	9 (22.50)	14 (40)		
Diabetes complications, n (%)				0.002 (1)	.97
Yes	13 (17.33)	7 (17.50)	6 (17.14)		
No	62 (82.67)	33 (82.50)	29 (82.85)		
Frequency of blood glucose monitoring, n (%)				13.4 (3)	.004
Never	10 (13.33)	6 (15)	4 (11.43)		
Occasionally	18 (24)	15 (37.50)	3 (8.57)		
Regularly	5 (6.67)	4 (10)	1 (2.86)		
Other	42 (56)	15 (37.50)	27 (77.14)		
Diabetes treatment, n (%)				4.6 (2)	.01
Insulin	23 (30.67)	8 (20)	15 (42.86)		
Insulin and oral medication	49 (65.33)	30 (75)	19 (54.29)		
Other	3 (4)	2 (5)	1 (2.86)		
Type of diabetes mellitus, n (%)				0.2 (2)	.89
Type 1	3 (4)	2 (5)	1 (2.86)		
Type 2	70 (93.33)	37 (92.50)	33 (94.29)		
Other types of diabetes	2 (2.67)	1 (2.50)	1 (2.86)		
Course of diabetes mellitus, n (%)				2.9 (4)	.09
≤1 month	41 (54.67)	16 (40）	25 (71.43)		
1‐6 months	11 (14.67)	7 (17.50)	4 (11.43)		
6 months-1 year	2 (2.67)	2 (5)	0 (0)		
1‐5 years	8 (10.67)	4 (10)	4 (11.43)		
>5 years	13 (17.33)	11 (27.50)	2 (5.71)		
Number of daily insulin injections, mean (SD)	2.64 (0.90)	2.63 (0.89)	2.66 (0.91)	−0.15 (73)	.88
Daily injection dose (U), mean (SD)	17.68 (9.64)	15.61 (9.04)	19.94 (9.89)	−1.96 (73)	.054
Insulin knowledge scores, mean (SD)	5.36 (2.23)	5.80 (2.13)	4.85 (2.28)	1.85 (73)	.07
Insulin attitude scores, mean (SD)	89.88 (15.47)	91.48 (17.11)	88.06 (13.37)	0.95 (73)	.34
Insulin practice scores, mean (SD)	27.55 (5.01)	27.57 (5.25)	27.33 (2.08)	0.08 (73)	.94

a BP: blood pressure.

b ¥1=US $0.14 as of December 30, 2022.

### Effect of the e-Coach Program on the Insulin Knowledge-Attitude-Practice Level

**Figure 3.**
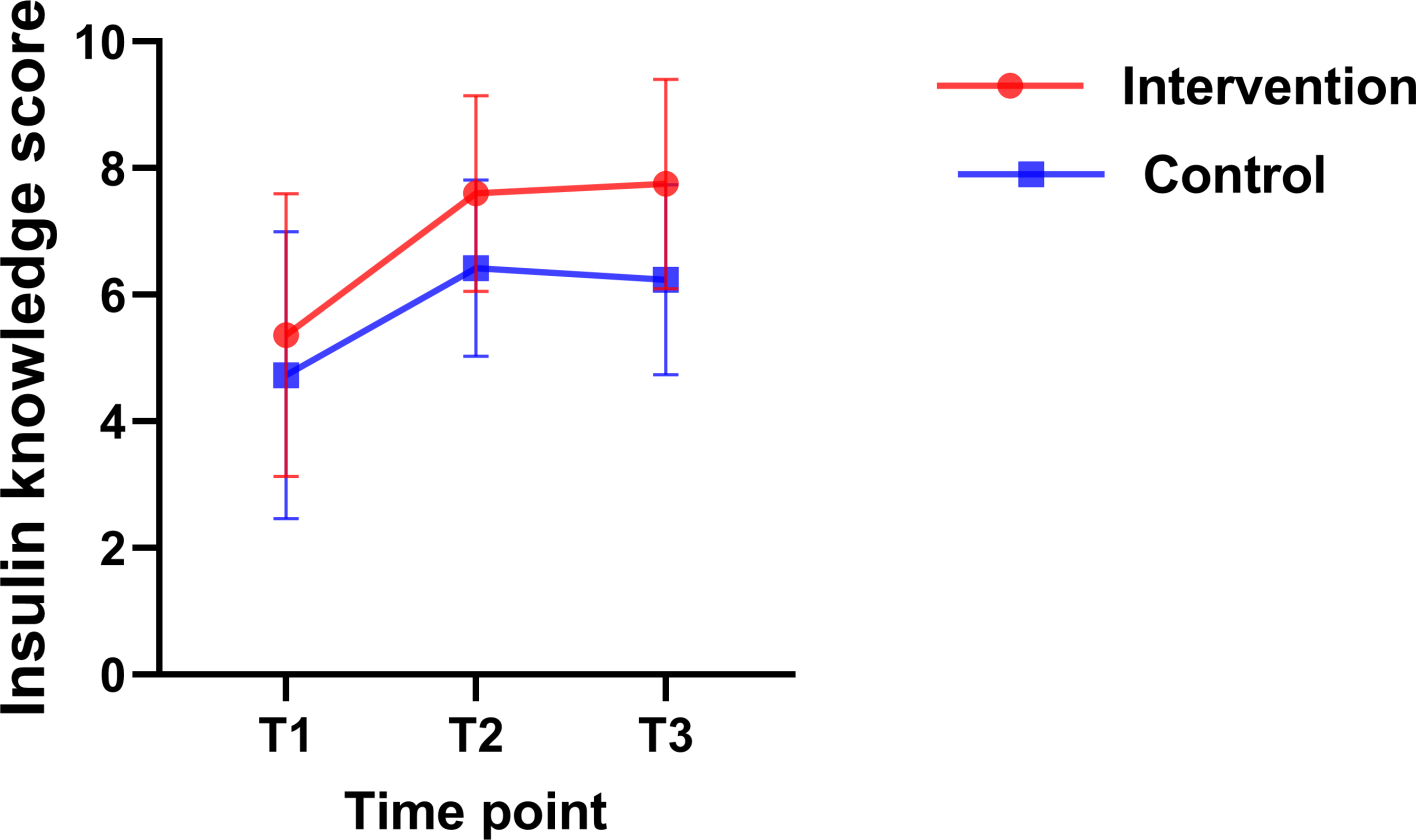
Mean (SD) insulin knowledge scores in intervention and control groups.

**Figure 4.**
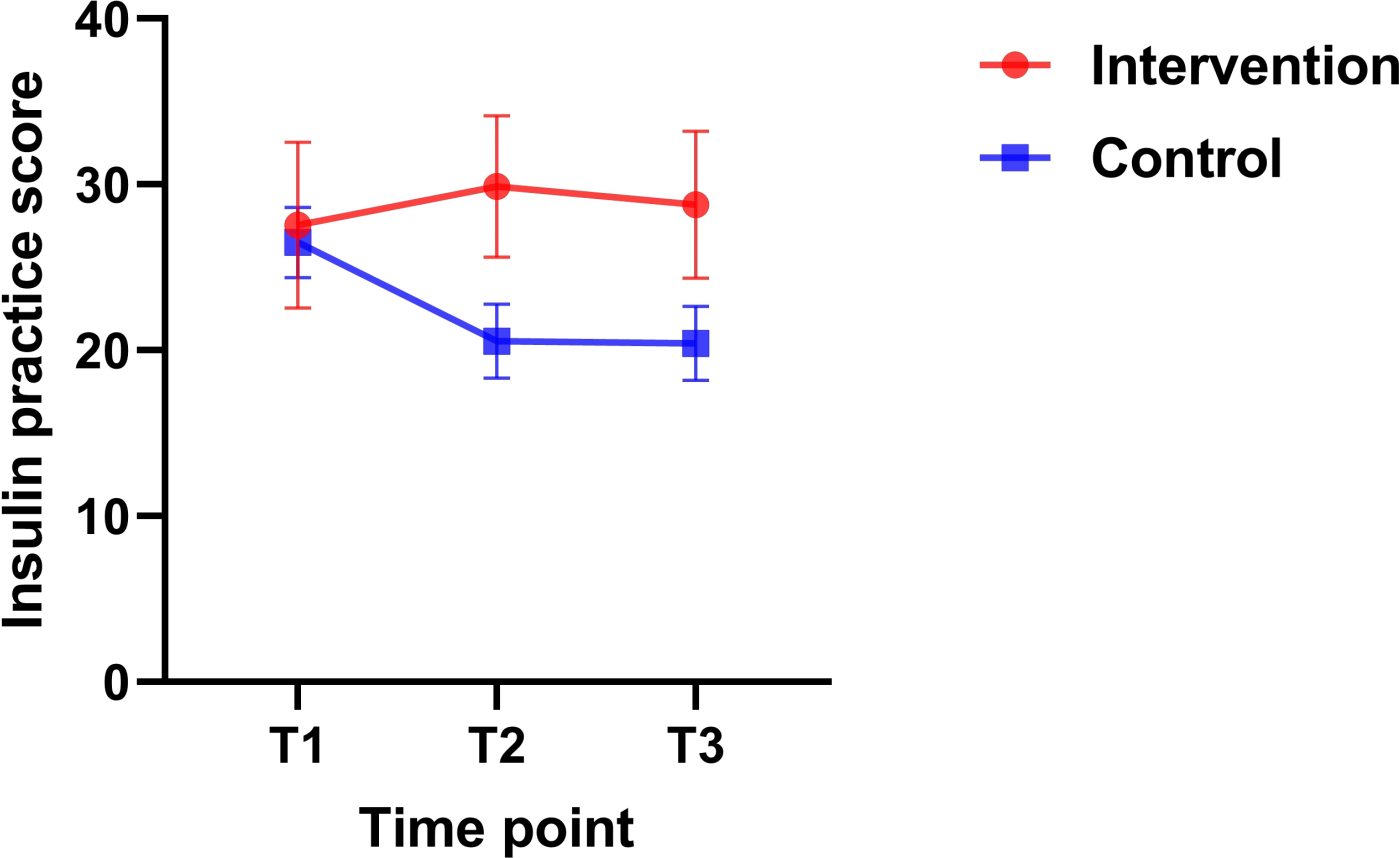
Mean (SD) insulin practice scores in intervention and control groups.

It was tested whether the insulin knowledge-attitude-practice scores obey an approximate normal distribution using chi-square test (*P*>.05; see Table 3 for exact *P* values), and the levels of insulin perception scores at different time points of the 2 groups of patients were analyzed using repeated-measures ANOVA, and the results of the sphericity test did not satisfy the conditions of Mauchly’s sphericity hypothesis, and the Greenhouse-Geisser test was used. The results (Table 4) showed statistically significant group × time interaction effects on total insulin knowledge scores (*P*=.03), but the group × time interaction effects on total insulin practice scores were not statistically significant (*P*=.08)—that is, the insulin knowledge of the 2 groups of patients at each time point showed an unequal tendency to change over time (Figure 3), but this difference was not obvious in insulin injection practice (*P=0.35*; Figure 4). The group factor could influence the total insulin knowledge-practice scores of the 2 groups of patients; pairwise comparisons showed that the total insulin knowledge score of the intervention group was higher than that of the control group at 1 and 3 months after the intervention, and the difference was statistically significant (*P*<.001).

**Table 4.** Repeated-measures ANOVA of the total insulin knowledge-attitude-practice scores in 2 groups at different time points.

Outcome	Time effect		Group effect		Interaction effect (time × group）	
	*F* test (*df*)	*P* value	*F* test (*df*)	*P* value	*F* test (*df*)	*P* value
Insulin knowledge scores	5.02 (2, 146)	.02	47.67 (1, 73)	<.001	4.75 (2, 146)	.03
Insulin practice scores	1.05 (2, 146)	.35	0.66 (1, 73)	.004	2.82 (2, 146)	.08
Insulin practice item 1	0.11 (2, 146)	.84	0.66 (1, 73)	.42	0.66 (2, 146)	.48
Insulin practice item 2	0.47 (2, 146)	.58	4.77 (1, 73)	.04	1.12 (2, 146)	.32
Insulin practice item 3	1.20 (2, 146)	.30	23.34 (1, 73)	<.001	0.61 (2, 146)	.52
Insulin practice item 4	1.39 (2, 146)	.26	1.94 (1, 73)	.18	2.36 (2, 146)	.14
Insulin practice item 5	2.30 (2, 146)	.13	8.35 (1, 73)	.007	5.06 (2, 146)	.02
Insulin practice item 6	1.66(2, 146)	.21	0.47 (1, 73)	.5	1.99 (2, 146)	.17
Insulin practice item 7	0.71 (2, 146)	.48	9.75 (1, 73)	.004	0.57 (2, 146)	.55
Insulin attitude scores	2.47 (2, 146)	.11	16.04 (1, 73)	<.001	12.52 (2, 146)	<.001
Insulin benefits	3.42 (2, 146)	.06	6.21 (1, 73)	.02	1.32 (2, 146)	.26
Insulin perception	0.82 (2, 146)	.39	7.37 (1, 73)	.008	18.86 (2, 146)	<.001
Life management	2.79 (2, 146)	.06	14.72 (1, 73)	<.001	10.34 (2, 146)	.001
Use attitude	2.79 (2, 146)	.09	14.72 (1, 73)	<.001	10.34 (2, 146)	<.001
Injection side effects	1.06 (2, 146)	.33	19.52 (1, 73)	<.001	4.62 (2, 146)	.02
Side effect	0.08 (2, 146)	.84	5.75 (1, 73)	.02	7.71 (2, 146)	.003
Cost	0.20 (2, 146)	.76	6.07 (1, 73)	.02	7.13 (2, 146)	.003

**Figure 5.**
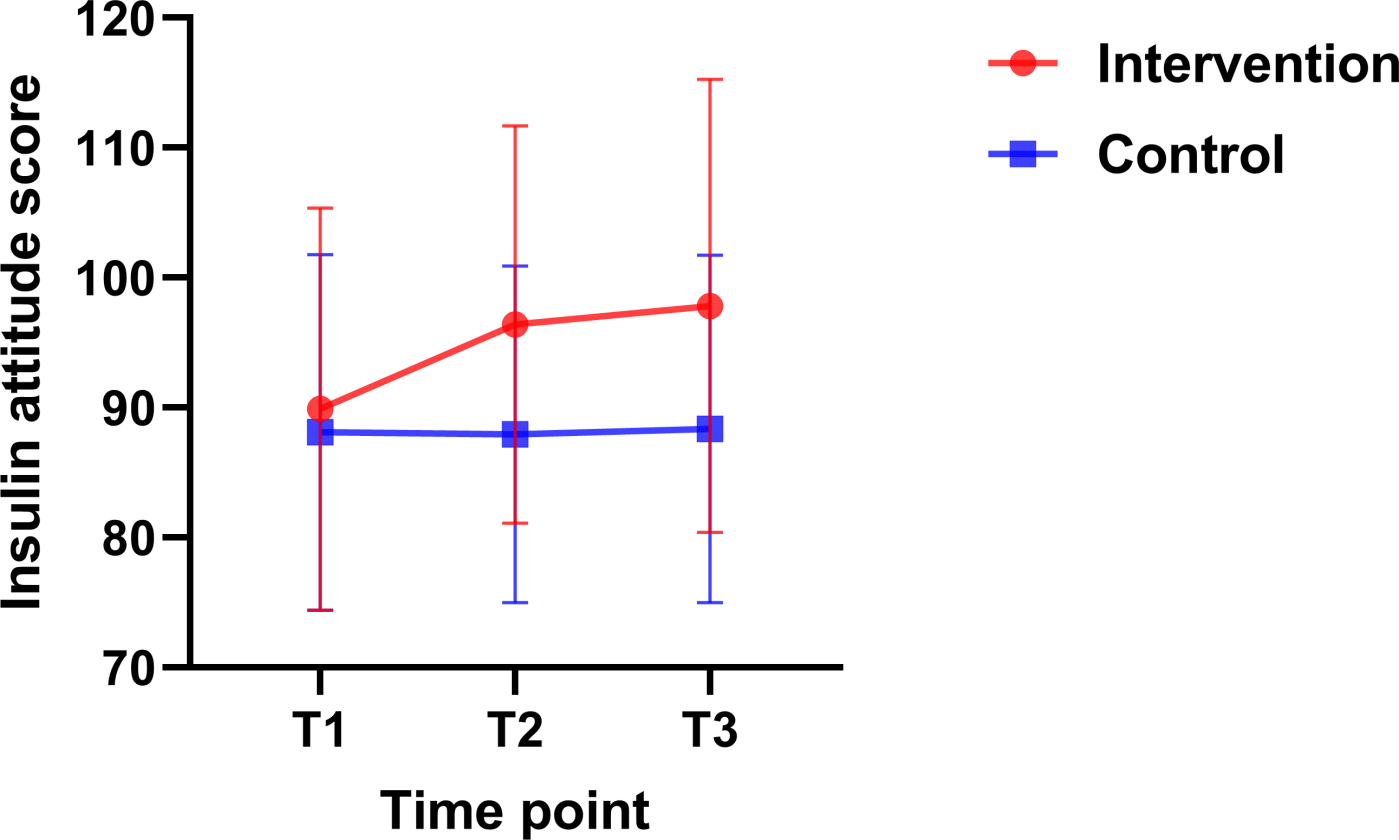
Mean (SD) insulin attitude scores in intervention and control groups.

The between-group effect (*P*<.001) and time × group interaction effect (*P*<.001) on the total insulin attitude scores of the 2 groups of patients were statistically significant—that is, the insulin attitude scores of the 2 groups of patients at various time points showed unequal trends over time (Figure 5), and the group factor could influence the insulin perception scores of the 2 groups of patients.

## Discussion

### Principal Findings

The findings of this study focused on the effectiveness of a TST-based e-coach program on the knowledge, attitude, and practice of patients self-administering insulin injections for the first time. The results of this study showed that the e-coach program is effective in improving insulin knowledge and insulin attitude but was not proven effective for insulin practice. This discrepancy may be attributed to several factors: the weak predictive power of self-regulatory variables for behavioral practice within the temporal event construct of the TST; the strong habitual nature of behavioral prepotency, which necessitates a longer intervention period; and patient-related psychological factors. A meta-analysis showed that the correlation between self-regulatory variables in TST and actual behavior was extremely low (*r*=0.04, 95% CI −0.07 to 0), whereas intention (*r*=0.33) and behavioral prepotency (*r*=0.38) exhibited only moderate correlations. Behavioral prepotency, which reflects the temporal cumulative effects of past behavior and habits, had a moderate influence on current behavior (*r*=0.38) [25]. Habit restructuring typically requires an intervention period exceeding 3 months; within a short-term framework, established behavioral patterns tend to override the practical translation of newly acquired cognitive gains. It has also been emphasized that, before applying TST to a specific behavioral domain, verifying the association between its regulatory variables and the target behavior is essential; otherwise, discrepancies between theoretical predictions and actual behavioral outcomes are likely to occur [26]. Furthermore, this may be related to the stigmatization experienced by patients initiating insulin therapy because of their disease, which makes them reluctant to seek medical help or adhere to treatment. A survey by Arifin et al [27] demonstrated that all 210 enrolled patients reported experiencing stigmatization and discrimination, and the degree of stigma exerted a significant negative impact on their quality of life, ultimately impeding effective disease management and public health efforts. In this study, the standardized insulin injection behavior validated by the e-coach model focused on enhancing users’ self-regulatory skills; its theoretical basis offered relatively weak explanatory power for behavior. Moreover, restructuring the injection habit itself requires an intervention period extending beyond 3 months and is difficult to correct through remote management alone. In addition, patients initiating insulin therapy may develop negative attitudes such as shame and perceived disease-related stigma, which can affect their quality of life and treatment adherence, thereby compromising the standardization of insulin injection behavior. Taken together, these possible contributing factors led to a discrepancy between knowledge, attitude, and insulin injection behavioral outcomes in this study, wherein cognitive gains could not be readily translated into stable behavioral practice within 3 months.

The results of this study showed that the reason for the low degree of improvement of insulin injection practice in the control group may be due to the lack of knowledge enhancement and time perspective [28]. Injection technique practice, as one of the factors affecting the blood glucose control of patients with diabetes with insulin injection,is vital in health education [29]. According to the TST, in addition to the social cognitive variables and self-regulation ability described by the TPB, TST should also include the time perspective (that is, the inconsistency in the length of time for which the intervention remains effective for a given individual leads to different value evaluation of individual practice) [28]. Studies have shown that many patients receiving insulin treatment, especially those who take insulin for the first time, tend to have a special fear of injection and lack of knowledge about diabetes prevention and management [30]. Due to the short duration of intervention in this study, it may not reach the degree of cognitive variables and time perspective potency of individual intention to change practice, that is, the improvement in the level of knowledge of patients in the control group may not be enough to change the direction of future practice, which led to the low improvement in insulin injection practice of patients. The prevention and control of chronic diseases requires early action before the symptoms of the disease appear; however, is it possible to adjust one’s practice in the appropriate direction only when they recognize the long-term consequences of current bad practice and try to reduce the intention-practice gap [31].

Furthermore, the results of item-specific scores for insulin injection behavior at different time points showed that the between-group differences were significant for item 3 (disinfecting an area >5 cm in radius centered on the injection site with alcohol) and item 5 (keeping the needle in place for more than 10 seconds after injection before withdrawal), and the interaction effect for item 5 was also significant. No statistically significant differences were observed for the remaining items. These findings suggest that the behavior of keeping the needle in place for more than 10 seconds after injection may be jointly influenced by the intervention and time factors, reflecting a dynamic change in patients’ mastery of this key point, which warrants reinforced emphasis during education. The degree of improvement in other behavioral aspects remains to be further enhanced, indicating that in patients receiving insulin injections for the first time, key weaknesses exist in procedural details such as preinjection assessment and injection site rotation. Merely reinforcing selected operational details is insufficient to avert all risks; individualized training must be implemented to strengthen these weaknesses [32]. In particular, for first-time insulin users, establishing a complete and standardized behavioral pattern at an early stage can prevent long-term complications such as lipohypertrophy, thereby further improving treatment adherence and quality of life.

Furthermore, the results of this study demonstrated that, after controlling for baseline covariates, the between-group differences in the temporal trends of insulin attitude scores across time points were not statistically significant. The TST underscores the predictive roles of domain specificity and temporal perspective on future health behaviors; however, the present findings did not reveal a significant trend of change in insulin attitudes over time among first-time insulin users. Studies [33,34] have shown that the most common reasons for the poor self-management ability of insulin-injecting patients are the lack of sufficient knowledge and motivation, and the lack of knowledge leads to nonstandard injection practice, which leads to complications. It is suggested that when guiding the insulin injection practice among patients initiating insulin therapy, medical staff should implement behavioral prepotency interventions targeting cognitive deficiencies as soon as possible after delivering the corresponding knowledge in the early stage of health education. The intervention contents may include the significance and benefits of health risk practices (misunderstanding of insulin injection process and consequences of nonstandard insulin injection) and health promotion practice (correctly standardizing insulin injection practice). To help patients adhere to the belief of connectivity between health practice and disease prevention, reduce the intention-practice gap, and narrow the gap between knowledge and health practice.

Repeated-measures ANOVA to test the effects of the e-coach health education management program on the insulin attitude of patients self-administering insulin injections for the first time showed significant differences in the intergroup effects and interaction effects between the 2 groups, indicating that group factors could affect the insulin attitude scores of the 2 groups. The insulin attitude scores of the 2 groups were not equal at each time point and showed the trend of changing with time. The TST emphasizes the predictive effect of domain-specific variables and time perspective on future health practice. The results of this study also show the changing trend of insulin attitude with time in patients self-administering insulin injections for the first time, which suggests that medical staff should comprehensively consider the 2-way influence of attitude cognition and time when paying attention to improving patients’ self-management ability so as to avoid fixed and stereotyped educational thinking. Health management attitudes regarding short-term and long-term benefits should be developed to enhance the influence of intention on future health practice [35]. As seen in Figure 5, the insulin attitude score of the test group showed an upward trend with time, while that of the control group was relatively stable, and the overall degree of change was not high. This indicates that the attitude of patients receiving insulin injections for the first time did not significantly improve since the end of routine care, and this information received less attention in daily life. It is recommended that nurses promote the concept of patients acquiring insulin knowledge and attitudes through future education to enhance their domain-specific knowledge and corresponding cognitive abilities.

The influence of patient age in this study should not be overlooked. The intervention population of the e-coach health education management program was restricted to first-time insulin users, whose overall age fell within the young and middle-aged range, making remote operation and management relatively easy. Consequently, the improvements in knowledge and attitude levels were reasonably favorable within the short-term intervention period. However, given the overall diabetes population, the proportion of older adults cannot be underestimated, and the extent to which their insulin-related knowledge, attitudes, and behaviors improve still requires further investigation. Previous results from the e-coach program have demonstrated that integrating one-on-one counseling with virtual group meetings reduced health coaches’ working hours by 40% without compromising the quality of implementation or the health benefits for older adults, thereby significantly enhancing project scalability and sustainability [36]. Moreover, home-based exercise programs remotely supervised via videoconferencing have been proven effective in overcoming mobility barriers, with systematic reviews supporting their feasibility and effectiveness in increasing physical activity participation among older adults [37,38]. For patients with chronic conditions such as obesity, diabetes, and hypertension, the e-coach intervention strategy, which is grounded in the “7 Steps to Health” approach and integrates self-determination theory, can further support the behavior change process [39]. Collectively, these findings indicate that the potential of e-coach health education program management in geriatric diabetes care merits further exploration. However, although older adults are progressively adapting to digital technologies, they still encounter difficulties in operating electronic health tools; therefore, intervention designs should emphasize age-friendly features such as simplified interfaces and voice interaction [32,40].

### Limitations

This study has limitations. It is worth further expanding the research on the intervention population. At present, the intervention population of the e-coach health education management program is limited to patients self-administering insulin injections for the first time, who are generally in the middle-aged and young age groups, and the remote operation and management is less difficult. Compared with the overall chronic disease population in China, the proportion of older patients remains substantial and should not be underestimated. Therefore, the extension and application of this intervention in the older patient population merit further exploration. In terms of intervention duration, it would be worthwhile to further validate the effects of interventions over 6 months or more to produce more accurate results.

### Conclusions

The e-coach program based on the TST was effective in improving insulin knowledge and insulin attitude but was not proven effective for insulin practice. Medical staff should comprehensively consider the 2-way influence of attitude cognition and time when paying attention to improving patients’ self-management ability, so as to avoid fixed and stereotyped educational thinking. Health management attitudes regarding short-term and long-term benefits should be developed to enhance the influence of intention on future health practice. With regard to the duration of the intervention, the effects may be more pronounced when the intervention lasts for 6 months or longer; it would be worthwhile to further investigate the extent of these long-term effects in the future.

## Supplementary material

10.2196/83339 Multimedia Appendix 1 Comparison of baseline characteristics between completers and dropouts within the intervention group and control group, respectively.

## Abbreviations

ANCOVA: analysis of covariance
BP: blood pressure
DAWN: Diabetes Attitude Wishes and Needs
TPB: theory of planned behavior
TST: temporal self-regulation theory
