# Supplementary material for: Effects of an e-Coach Program on the Knowledge, Attitude, and Practice of Patients Self-administering Their First Insulin Injection: Quasi-Experimental Study
Source: JMIR Form Res. 2026 Jul 10;10:e83339. doi: 10.2196/83339 (PMC13359486; doi:10.2196/83339)
Supplement: Multimedia Appendix 1 [file formative-v10-e83339-s001.docx]

**Supplementary Table S1** Comparison of Baseline Characteristics between Completers and Dropouts within the Intervention Group (n=43)

| **Characteristics** | | | **Completers（n=40）** | **Dropouts（n=3）** | **test statistic value** | ***p*** |
| --- | --- | --- | --- | --- | --- | --- |
| Gender | |  |  |  |  |  |
|  | Male |  | 30（69.77） | 2（4.65） | 0.102 | 0.750 |
|  | Female |  | 10（23.26） | 1（2.33） |  |  |
| Age, years | | | 46.15±12.06 | 43.33±11.15 | 0.392 | 0.697 |
| Height, (cm) | |  | 169.33±7.32 | 163.67±12.22 | 1.239 | 0.222 |
| Weight, (kg) | |  | 72.90±16.93 | 62.33±12.50 | 1.054 | 0.298 |
| BP, (mmHg) | |  |  |  |  |  |
| Systolic pressure | |  | 125.45±15.48 | 127.67±7.51 | -0.244 | 0.809 |
| Diastolic pressure | |  | 81.40±10.31 | 74.67±5.86 | 1.110 | 0.273 |
| Marital status | |  |  |  |  |  |
|  | Single |  | 34（79.07） | 3（6.98） | 0.523 | 0.470 |
|  | Married |  | 6（13.95） | 0（0.00） |  |  |
| Monthly income | |  |  |  |  |  |
|  | ＜3000 |  | 5（11.63） | 1（9.30） | 1.544 | 0.462 |
|  | 3000-5000 |  | 9（20.93） | 0（0.00） |  |  |
|  | ＞5000 |  | 26（60.47） | 2（4.65） |  |  |
| Education level | |  |  |  |  |  |
|  | Junior high school and below | | 16（37.21） | 2（4.65） | 1.600 | 0.449 |
|  | Senior high school or college degree | | 14（32.56） | 0（0.00） |  |  |
|  | Bachelor degree or above | | 10（23.26） | 1（2.33） |  |  |
| Occupation | |  |  |  |  |  |
|  | Workers or farmers | | 10（23.26） | 0（0.00） | 2.033 | 0.730 |
|  | Staff |  | 17（39.53） | 1（2.33） |  |  |
|  | Retirees |  | 6（13.95） | 1（2.33） |  |  |
|  | Students |  | 1（2.33） | 0（0.00） |  |  |
|  | Others |  | 6（13.95） | 1（2.33） |  |  |
| Diabetes complications | |  |  |  |  |  |
|  | Yes |  | 5（11.63） | 1（2.33） | 1.009 | 0.315 |
|  | No |  | 35（81.40） | 2（4.65） |  |  |
| Frequency of blood glucose monitoring | | | | | | |
|  | Never |  | 6（13.95） | 0（0.00） | 1.204 | 0.752 |
|  | Occasionally |  | 17（39.53） | 2（4.65） |  |  |
|  | Regularly |  | 5（11.63） | 0（0.00） |  |  |
|  | Others |  | 12（27.91） | 1（2.33） |  |  |
| Type of diabetes mellitus | | |  |  |  |  |
|  | Type 1 diabetes mellitus | | 1（2.33） | 0（0.00） | 0.157 | 0.924 |
|  | Type 2 diabetes mellitus | | 38（88.37） | 3（6.98） |  |  |
|  | Other types of diabetes | | 1（2.33） | 0（0.00） |  |  |
| Course of diabetes mellitus | | | | | | |
|  | ≤1 month |  | 13（30.23） | 1（2.33） | 2.617 | 0.624 |
|  | 1-6 month |  | 8（18.60） | 0（0.00） |  |  |
|  | 6 month-1 year |  | 2（4.65） | 0（0.00） |  |  |
|  | 1-5 years |  | 6（13.95） | 0（0.00） |  |  |
|  | ＞5years |  | 11（25.58） | 2（4.65） |  |  |

**Supplementary Table S2** Comparison of Baseline Characteristics between Completers and Dropouts within the Control Group (n=43)

| **Characteristics** | | | **Completers（n=35）** | **Dropouts（n=8）** | **test statistic value** | ***p*** |
| --- | --- | --- | --- | --- | --- | --- |
| Gender | |  |  |  |  |  |
|  | Male |  | 29（67.44） | 5（11.63） | 1.631 | 0.202 |
|  | Female |  | 6（13.95） | 3（6.98） |  |  |
| Age, years | | | 42.06±13.81 | 35.00±14.23 | 1.297 | 0.202 |
| Height, (cm) | |  | 171.37±8.37 | 173.13±8.76 | -0.530 | 0.599 |
| Weight, (kg) | |  | 80.43±17.12 | 85.00±22.12 | -0.645 | 0.523 |
| BP, (mmHg) | |  |  |  |  |  |
| Systolic pressure | |  | 133.29±24.10 | 123.88±27.78 | 0.970 | 0.338 |
| Diastolic pressure | |  | 89.11±16.42 | 82.63±16.87 | 1.004 | 0.321 |
| Marital status | |  |  |  |  |  |
|  | Single |  | 24（55.81） | 5（11.63） | 0.000 | 1.000 |
|  | Married |  | 11（25.58） | 3（6.98） |  |  |
| Monthly income | |  |  |  |  |  |
|  | ＜3000 |  | 2（4.65） | 0（0.00） | 1.293 | 0.524 |
|  | 3000-5000 |  | 3（6.98） | 0（0.00） |  |  |
|  | ＞5000 |  | 20（46.51） | 8（18.60） |  |  |
| Education level | |  |  |  |  |  |
|  | Junior high school and below | | 11（25.58） | 0（0.00） | 4.232 | 0.121 |
|  | Senior high school or college degree | | 14（32.56） | 5（11.63） |  |  |
|  | Bachelor degree or above | | 10（23.26） | 1（2.33） |  |  |
| Occupation | |  |  |  |  |  |
|  | Workers or farmers | | 3（6.98） | 0（0.00） | 8.981 | 0.062 |
|  | Staff |  | 16（37.21） | 1（2.33） |  |  |
|  | Retirees |  | 3（6.98） | 0（0.00） |  |  |
|  | Students |  | 0（0.00） | 1（2.33） |  |  |
|  | Others |  | 13（30.23） | 4（9.30） |  |  |
| Diabetes complications | |  |  |  |  |  |
|  | Yes |  | 5（11.63） | 3（6.98） | 2.197 | 0.138 |
|  | No |  | 30（69.78） | 3（6.98） |  |  |
| Frequency of blood glucose monitoring | | | | | | |
|  | Never |  | 4（9.30） | 0（0.00） | 1.704 | 0.636 |
|  | Occasionally |  | 3（6.98） | 0（0.00） |  |  |
|  | Regularly |  | 1（2.33） | 0（0.00） |  |  |
|  | Others |  | 27（62.79） | 6（13.95） |  |  |
| Type of diabetes mellitus | | |  |  |  |  |
|  | Type 1 diabetes mellitus | | 1（2.33） | 0（0.00） | 0.157 | 0.924 |
|  | Type 2 diabetes mellitus | | 38（88.37） | 3（6.98） |  |  |
|  | Other types of diabetes | | 1（2.33） | 0（0.00） |  |  |
| Course of diabetes mellitus | | | | | | |
|  | ≤1 month |  | 24（55.81） | 4（9.30） | 2.617 | 0.624 |
|  | 1-6 month |  | 5（11.63） | 1（2.33） |  |  |
|  | 6 month-1 year |  | 0（0.00） | 0（0.00） |  |  |
|  | 1-5 years |  | 4（9.30） | 0（0.00） |  |  |
|  | ＞5years |  | 2（4.65） | 1（2.33） |  |  |
